# Current situation and future development of the biopharmaceutical industry in China: A mixed-method study

**DOI:** 10.3389/fphar.2022.911165

**Published:** 2022-08-22

**Authors:** Ruomeng Yang, John Alimamy Kabba, Xuelin Yao, Caijun Yang, Jie Chang, Wenjing Ji, Minghuan Jiang, Mingyue Zhao, Jun Wen, Yu Fang

**Affiliations:** ^1^ Department of Industrial Economics and Trade, School of Economics and Finance, Xi’an Jiaotong University, Xi’an, China; ^2^ Department of Pharmacy Administration and Clinical Pharmacy, School of Pharmacy, Xi’an Jiaotong University, Xi’an, China; ^3^ Center for Drug Safety and Policy Research, Xi’an Jiaotong University, Xi’an, China; ^4^ Shaanxi Centre for Health Reform and Development Research, Xi’an, China

**Keywords:** biopharmaceutical, biotechnology, biosimilars, China, innovation, biological medicine

## Abstract

**Introduction:** The biomedical industry has grown significantly both globally and in China; however, there are still challenges. This study aimed at evaluating the biopharmaceutical sector of China, in terms of ability to innovate, current sales volume, investment, and R&D expenditure, as well as providing a case study detailing the progress and challenges of the industry in Shaanxi province.

**Method:** A cross-sectional mixed-method study design was used to generate a comprehensive profile of the nature of biopharmaceutical innovation capacity and development in China by triangulating country-wide survey and interview data from Shaanxi province. Only biopharmaceutical companies that are currently marketing or conducting research and development were eligible for inclusion, and Shaanxi province was selected for conducting the interviews. Categorical and continuous variables were analyzed descriptively. Interviews were thematically analyzed by using NVivo version 12.

**Results:** The analysis includes responses from 77 biopharmaceutical enterprises; the majority (36, 46.8%) are in Eastern China, followed by 26 (33.8%) in Central China. In 2018, the total sales of biological products amounted to 26.28 billion yuan, and in 2019, a slight increase was observed (30.34 billion); the amount doubled in 2020 to about 67.91 billion yuan. The top three biopharmaceutical products on sale in 2020 were Camrelizumab (5.14 billion yuan), human albumin (4.56 billion yuan), and human immunoglobulin for injection (3.78 billion yuan). Expenditure on R&D has also increased; it amounted to 1657.7 million yuan in 2018, which more than doubled in 2019 to 3572.1 million yuan and further increased to 5857.7 million yuan in 2020. Nonetheless, the progress is not uniform across all provinces, as shown from the results from Shaanxi province, because of lack of local government policies that will impact on the funding, incentives, and market share that motivate the producers.

**Conclusion:** China’s biopharmaceutical industry has expand significantly. The increase in sales indicates that there is an increase in demand for biologicals; moreover, R&D funding is increasing. These are key indicators that influence innovation and development. However, the sector’s capacity to innovate and develop needs to be improved, particularly in the western region, where research and production are relatively weak.

## 1 Introduction

The biomedical industry has made considerable progress in the past few decades since its inception in the 1970s. These achievements are largely the result of a shift in production from traditional chemical drugs to innovative biological and large molecules (Zhang and Liu, 2020). Today, many previously inconceivable or incurable diseases can be effectively diagnosed and prevented or treated by using innovative diagnostics and biologicals, respectively (Kesik-Brodacka, 2018; Cutler et al., 2020; Uppal et al., 2020). Biopharmaceuticals are pharmaceuticals with biological entities as their ingredients, manufactured by using biotechnology methods that may be new breakthrough technologies or older techniques (Rader, 2008). Biopharmaceuticals represent one of the best accomplishments in the 21st century, partly because of their superiority in terms of treatment outcomes and management of diseases, with small to negligible side effects relative to those of chemical products (Kesik-Brodacka, 2018; Kinch et al., 2021). Moreover, they exert a higher degree of activity and specificity than traditional drugs (Kesik-Brodacka, 2018). For most nations, progress in biopharmaceutical capabilities is a national agenda, a source of pride, and a testament of scientific progress (Schmid and Xiong, 2021; Zhang and Liu, 2020). However, biologicals are complex molecules requiring enormous resources (human, capital, and others) and the industry is rigorously regulated; therefore, maintaining progress and attaining the economic benefits required for sustained innovations in both product and process is key (Uppal et al., 2020).

The United States of America (USA), along with European nations, pioneered the industry and have been leading ever since in terms of capital investment, infrastructure, research and development, and market shares, thus more or less dictating the dynamics of the sector (Freire et al., 2020; Zhang and Liu, 2020). China in recent times has emerged as a key global economic player, making tremendous progress in science and technology (S&T), and is set to overhaul its biopharmaceutical industry in a trajectory that competes with or leapfrogs previous major players, including the United States, Europe, and Japan, probably by the coming decade (Freire et al., 2020). Chinese investment in S&T is estimated to be about 2.2% of GDP, and China has mapped out policies that aggregate multiple fronts toward the ambition to lead the sector in magnitude and innovation (Schmid and Xiong, 2021; Atkinson, 2020).

China locally developed and marketed its first biopharmaceutical product, recombinant human interferon alpha 1b, in 1989, marking progress in the industry that started in the early 1980s. Since then, China has ambitiously embarked on what can be described as multifaceted strategic developmental endeavors, including enacting reforms and policies, constructing research and development centers, and overhauling its drug regulatory framework with the overall objective of sustained development of the Chinese biopharmaceutical sector (Tripathy et al., 2020; Zhang and Liu, 2020). Between 1989 and 1995, three state-sponsored R&D centers with the capacity to develop biopharmaceutical products, which included centers for genetically engineered drugs, biological products, and vaccines, were established (Zhang and Liu, 2020). What followed onward until 2000 was the implementation of the “1035 Plan” by the Ministry of Science and Technology, aimed at promoting the R&D of new medicinal entities. The “Medical Science and Technology Policy (2002010),” “Bio-Industry Development Eleventh Five-Year Plan,” and “Made in China 2025” are all geared toward supporting and expanding the capability of the biopharmaceutical landscape (Hu et al., 2015; Atkinson, 2020; Zhang and Liu, 2020).

With all these inputs, the industry has developed significantly in the past 3 decades in magnitude and capacity, attaining the second largest biopharmaceutical market globally in 2019 (Tripathy et al., 2020). Growth has been consistent; for instance, the Chinese biopharmaceutical market grew from 4.5% in 2010 to 15.3% in 2017, equivalent to a market size of 341.171 billion Chinese yuan (Zhang and Liu, 2020), and it is forecast to exceed that of the United States by 2022 (Gu, 2021b; Distlerath, 2021). Additionally, as of 2017, there were an estimated 800 innovative molecules in different stages of development (preclinical and clinical trials) in China. There has also been an increase in the R&D of innovative drugs since 2017. For example, a Chinese enterprise was reported to be planning to start three clinical trials of drugs developed by using the CRISPR technique for treatments that target bladder, prostate, and renal cell cancers (Atkinson, 2020). In 2018, about 25 Chinese companies filed applications for PD-1/PD-L1 inhibitors, advanced anticancer biomolecules, and in 2019, close to 40% of companies conducting clinical trials in chimeric antigen receptor treatment (CAR-T) were Chinese companies (Atkinson, 2020; Kesik-Brodacka, 2018). At the start of the current COVID-19 pandemic, China was swift in sequencing the viral genome, which helped global and local biopharmaceutical manufacturers in the development of vaccines against the virus (Cheng et al., 2021), an achievement that spoke volumes about the transformation of the Chinese biopharmaceutical industry.

Amid this progress, the Chinese pharmaceutical sector is mostly dominated with generic and traditional Chinese medicines (TCM) (Schmid and Xiong, 2021) and it is relatively immature. In terms of its biopharmaceutical industry, recent global reports by the Biopharmaceutical Competitiveness & Investment Survey (BCI), assessing the extent of scientific and clinical capabilities, regulatory framework, access to market, and intellectual property (IP) environment, as well as recent pertinent policy issues in the given group of markets, categorized the Chinese biopharmaceutical market as a “newcomer” (PHRMA, 2017). Furthermore, compared with global leading players, the sector is less competitive, and it is challenged by weak innovative capacity, low R&D investment (Chan et al., 2016), and the fact that progress is not decentralized across China (Schmid and Xiong, 2021). A large proportion of highly innovative drugs still comes from non-Chinese biopharma (Schmid and Xiong, 2021), and even though China dominates the generic drug market, it has yet to manifest itself in the biosimilar drug market, which is considered to be relatively small with progress slowing down (Zhang and Liu, 2020). Even so, there is a lack of current research on the latest status of the biopharmaceutical industry in China and of a case study focusing on Shaanxi province specifically. This study was aimed at assessing the capacity of China’s biopharmaceutical industries to innovate and the current developments in terms of sales volume, investment, and R&D expenditure with a case study highlighting the progress and challenges of the industry in Shaanxi province. To attain the underlying objective, the discussion focused on the top portfolio of products, regional concentration of companies within mainland China, market capitalization of key products, sales forecasts, investment and expenditure in R&D, and products in development.

## 2 Materials and methods

### 2.1 Study designs

In this study we employed a cross-sectional design, triangulating data from both a national survey and a semi-structured interview in Shaanxi province to generate a comprehensive profile of the nature of biopharmaceutical innovation capacity and development in China. This approach is necessary because the two methods complement the strength of the findings and reduce the limitations that might be associated with a single research method (Sawatsky et al., 2019).

### 2.2 Data collection tools

Two data collection tools were developed in this research; a questionnaire and an interview guide. In developing the questionnaire, we followed a three-stage process described previously (Xu et al., 2018). Firstly, the research team outlined the study objective and keywords that were used to search the relevant literature. Articles (Schmid and Xiong, 2021; Hu et al., 2015, Atkinson, 2020; Tripathy et al., 2020) that were within the scope of the study were screened and sorted, and a preliminary document was drafted. Lastly, the draft was reviewed by the research team and sent to two specialists in the biopharmaceutical industry in China for their inputs and professional screening for readability and appropriateness to the study objectives. The comments from the experts and research team informed the final questionnaire used in data collection. In brief, the questionnaire contained items that assessed company characteristics including the type of enterprise, the number of employees, and their designation and educational level. It also evaluated the name and revenue generated by the top five biopharmaceutical drugs and the expenditure on R&D in the past 3 years from 2018-2020. Furthermore, an item was included that assessed details of the innovative projects undertaken and proposed in the past 3 years and next 5 years, respectively, including the type, level of development, and estimated funding amount. Finally, a question on the type of services that the company wishes to out-source to achieve its developmental plans was explored (see Supplementary Appendix S1).

For a detailed elaboration from the perspective of the biopharmaceutical companies on factors that have been highlighted in the literature as impediments to the development and innovation of the biopharmaceutical industry in China, we conducted a semi-structured interview in Shaanxi province. Interviews were targeted at departmental managers of four pharmaceutical companies in Shaanxi province. Shaanxi province was selected firstly because it is among the provinces in China that are considered to have a poorly developed biopharmaceutical industry, it produces fewer products (about 1-2 per company), and the companies have lower innovation capacity in comparison with companies in other provinces in China (Zhang and Liu, 2020). Secondly, the affiliated institution that conducted this study is located in Shaanxi; hence, it was possible for us to understand from a company perspective the main challenges affecting the development of the industry and provide targeted recommendations. Lastly, the recruitment of participants was less challenging than it might have been if the researchers had included other provinces with the same characteristics as Shaanxi province. The interview guide used for the interviews was developed after consultations with key experts in the pharmaceutical policy units and members of the biopharmaceutical association in Shaanxi province. This approach was followed because there is no report in the published literature on the topic. In brief, the interview guide had six main items, including general questions on the most important challenges and the impact of national drug negotiations policy and the national centralized procurement policy toward company development. Furthermore, one item probed the dynamics and impacts of excluding innovative biopharmaceutical drugs from national medical insurance on current and future R&D (see Supplementary Appendix S2 for details).

### 2.3 Sampling method and process

There is ambiguity in what is defined as biotechnology in China; thus, the exact number of biopharmaceuticals is difficult to ascertain. According to “The China Pharmaceutical Innovation and Research Development Association,” there is an estimated domestic pharmaceutical sector comprised of over 4000 companies, but only a few of them are currently engaged in R&D or sales of biopharmaceuticals products (2019). Therefore, for this research, only biopharmaceutical companies currently marketing or conducting R&D for at least one product were eligible for inclusion. After consultation with the China Biomedical and Pharmaceutical Industry Association, we developed a convenient sampling frame with the following criteria. Companies with a reachable executive holding a top management position in the past 3 years who had a detailed understanding of the enterprise and was willing to participate in the study were contacted. We adopted this approach because it is difficult for companies to grant interview requests on the sensitive issues that we wish to explore. For the survey, a list of the selected companies and their contact information was generated. The researchers sent emails to all eligible companies explaining the objectives of the study and how their data would be used. A follow-up reminder call was made to those who did not respond within three working days. The companies that consented to take part in the study were sent a link to the online questionnaire. A similar contact process was used to recruit participants from Shaanxi province for the interviews.

### 2.4 Data collection

The two data types, interview and survey, were collected simultaneously and online owing to the current epidemic situation. Each participant was given a detailed consent form outlining the study’s objectives and how the data would be handled. Furthermore, we assured participants that their information would be kept private and that they could decline at any time during the data collection process. A link to the questionnaire was sent to the company by email or WeChat, and responses were received automatically in a repository. All interviews were conducted by lead researchers with the right competence and experience in conducting interviews in Chinese after a pre-arranged agreement with the interviewee via telephone calls, and the conversations were recorded by using a voice recorder. Data collection lasted from 2021-08-15 to 2021-10-31.

### 2.5 Data management and analysis

Survey data were exported to MS Excel for cleaning, analysis, and visualization. Categorical and continuous variables were analyzed descriptively by using summation, frequency, and proportion. For the qualitative data, two members of the research team transcribed the interviews verbatim in MS Word, and then both transcripts were reconciled by the whole research team for accuracy and completeness. The transcribed interviews were deidentified and then translated from Chinese to English for analysis. Following an inductive analytical approach, the transcripts were thoroughly read by the lead authors; the texts were mapped into themes and further categorized into general and subthemes, as per best practices reported previously (Colorafi and Evans, 2016). The other members of the research team reviewed the results from the two leading authors, and a consensus on the themes was met, which informed the final result. Interview data were managed and analyzed by using NVivo version 12.

### 2.6 Ethical clearance

This study was approved by Xi’an Jiaotong University’s Ethics Committee; document approval number (No): 2022-1406.

## 3 Results

### 3.1 Characteristics of enterprises

All companies contacted responded to the online questionnaire, amounting to a total of 83 responses, of which three were duplicated; another three enterprises reported no biopharmaceutical product on sale or under development and hence were excluded from the statistical analysis. The analysis included responses from 77 biopharmaceutical enterprises, with the majority (36) from Eastern China (46.8%), 26 from Central China (33.8%), and 15 from Western China (19.5%). At the provincial level, Guangdong (10, 13.0%) had the highest concentration of companies followed by Jiangsu (9, 11.7%). Most of the companies (70, 90.9%) are categorized as high-tech, and 75 (97.4%) have established independent research and development departments (see details in Table 1) In terms of biopharmaceutical enterprise employees, the results show that 5406 (5.6%) are highly educated with MSc and PhD qualifications; another 244 (0.25%) are dedicated intellectual property management staff (see Table 2).

**TABLE 1:** Geographic distribution of biopharmaceutical enterprises included in this study.

Region	Province	Freq. (%)	High-tech enterprises	Independent R&D departments
Eastern	Guangdong	10 (13.0)	8	10
	Jiangsu	9 (11.7)	8	9
	Shandong	4 (5.2)	4	4
	Hebei	4 (5.2)	4	4
	Zhejiang	3 (3.9)	3	3
	Liaoning	3 (3.9)	3	3
	Beijing	1 (1.3)	1	1
	Shanghai	1 (1.3)	1	1
	Hainan	1 (1.3)	1	1
Central	Hunan	6 (7.8)	6	6
	Heilongjiang	5 (6.5)	4	4
	Henan	4 (5.2)	3	4
	Jilin	4 (5.2)	3	4
	Hubei	3 (3.9)	3	3
	Jiangxi	2 (2.6)	2	2
	Anhui	1 (1.3)	1	1
	Shanxi	1 (1.3)	1	1
Western	Sichuan	6 (7.8)	6	6
	Shaanxi	5 (6.5)	4	5
	Guizhou	2 (2.6)	2	1
	Chongqing	1 (1.3)	1	1
	Yunnan	1 (1.3)	1	1

**TABLE 2:** Distribution of pharmaceutical personnel and the level of development of products according to provinces in China.

Region	Province	Total employees	Personnel			IP staff	Number of products	Development state of products in the past 5 years				In production
			PhD/MSc degree	Intermediate management position	Senior management position			Preclinical	Clinical approval	Clinical trial	Production certificate or new drug certificate	
Eastern	Guangdong	19601	1444	1411	46	59	12	4	1	4		3
	Jiangsu	9185	1153	256	50	28	12	2		4	1	5
	Shandong	6560	330	159	30	23	4	2		1		1
	Hebei	32557	1232	436	124	28	4			4		
	Liaoning	477	21	6	3	2	3	1	1	1		
	Beijing	2440	166	120	11	12	3	1	2			
	Shanghai	316	12	10	3	1	2			1		1
	Zhejiang	993	61	41	23	1	1	1				
Central	Jiangxi	700	37	26	9	1	7	5		1		1
	Jilin	6472	233	289	59	28	6	2		3		1
	Henan	2781	142	107	50	4	3	3				
	Hunan	1561	8	6	2	3	3	1	1	1		
	Hubei	4614	134	93	43	9	2	1		1		
	Shanxi	440	38	20	10	2	2			2		
	Heilongjiang	620	24	16	9	7	1	1				
	Anhui	1500	126	44	15	6	1			1		
Western	Sichuan	885	48	103	27	0	7	6				1
	Guizhou	3035	137	82	34	18	2	1		1		
	Chongqing	1869	54	77	34	10	2			2		
	Yunnan	469	6	10	7	2	1			1		

### 3.2 Biopharmaceutical sales for 2018-2020

**FIGURE 1:**
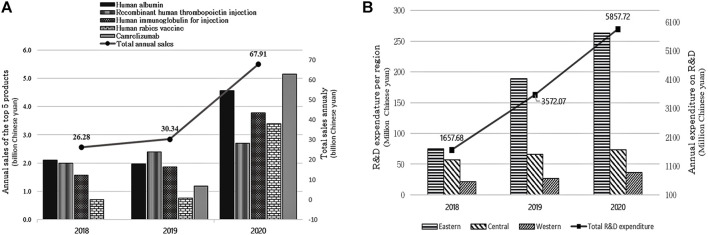
**(A)** shows the total sales of the top five biopharmaceutical drugs for 2018–2020 and **(B)** shows the expenditure in R&D for 2018–2020.

Biopharmaceutical product sales have grown during the last 3 years. For example, overall sales in 2018 were 26.28 billion yuan, with a small increase in 2019 (30.34 billion yuan), and sales revenue climbed dramatically to almost 67.91 billion yuan in 2020 (123.8% increase). The top three biopharmaceutical products on sale in 2020 were Camrelizumab (5.14 billion yuan), human albumin (4.56 billion yuan), and human immunoglobulin for injection (3.78 billion yuan). In 2019 and 2018, the top three products with the highest sales were recombinant human thrombopoietin injection (2.4 billion yuan in 2019 vs. 2 billion yuan in 2018), human albumin (1.97 billion yuan in 2019 vs. 2.1 billion yuan in 2018), and human immunoglobulin for injection (1.86 billion yuan in 2019 vs. 1.57 billion yuan in 2018) (see Figure 1A).

### 3.3 Annual R&D expenditure on biopharmaceuticals for 2018-2020

Figure 1B shows trends in R&D expenditure in the 3 years from 2018 to 2020. Total annual expenditure on R&D has been increasing. For instance, 1657.7 million yuan was spent on R&D in 2018; that figure more than doubled in 2019 to 3572.1 million yuan and further increased to 5857.7 million yuan in 2020. Most of the funding came from within the enterprises and the rest from government grants, subsidies, and bank loans. In comparing regional R&D expenditure, companies in the eastern part of China allocated the highest funds in the past 3 years followed by those in the central region. The western region spent the least on R&D.

### 3.4 Technology projects and products in development

As shown in Table 3, 48 projects involving the development of biopharmaceutical drugs were ongoing in the past 3 years, as reported by 17 of the surveyed enterprises. Most of the projects (28) were independent projects being undertaken by the respective companies but also included were commissioned projects (11 projects), joint ventures (8 projects), and technology transfer (1 project). Sichuan province (22 projects) in the west of China reported the highest number of projects in the past 3 years. The eastern and central enterprises each reported 13 ongoing projects of different types.

**TABLE 3:** Distribution of the number of projects in progress in the past 3 years.

Region	Province	Type of projects in progress in the past 3 years			
		Independent enterprise projects	Commissioned development projects	Cooperative development projects	Technology transfer project
Eastern	Guangdong	6		2	
	Jiangsu	2		1	
	Shandong	1			
	Hebei				
	Liaoning				
	Beijing			1	
	Shanghai				
	Zhejiang				
Central	Jiangxi	1			
	Jilin		1	1	
	Henan	1		1	
	Hunan	1	1		
	Hubei				
	Shanxi			1	1
	Heilongjiang	1	1		
	Anhui	1	1		
Western	Sichuan	14	7	1	
	Guizhou				
	Chongqing				
	Yunnan				

Furthermore, 47 companies reported a total of 78 new biopharmaceutical products, with the eastern region accounting for more than half (41, 52.5%). The remainder were from the central (25, 32.1%), and western (12, 15.4%) regions of China. Table 2 shows details of products that have been in production and marketing for the past 5 years. The highest proportion of these products (31, 39.7%) are in the preclinical stage of development; additionally, 26 (33.3%) are in clinical trials, 13 (16.7%) are in production, 5 (6.4%) have been clinically approved, and only 3 (3.8%) have obtained production certificates or new drug certificates. The products include Bevacizumab, insulin glargine injection, Dalteparin sodium injection, quadrivalent influenza virus split vaccine, *Haemophilus* influenzae type b conjugate vaccine, human fibrinogen, human coagulation factor VIII, and human prothrombin complex, which are at various stages of development and marketing in different provincial regions in China (Table 4).

**TABLE 4:** Products in development in multiple biopharmaceutical enterprises.

Product	Province	Proposed investment (millions)	Investment made (millions)	R&D stage
Bevacizumab	Guangdong	50.0	13.0	Clinical approval
	Jiangsu	-	-	Production
Insulin glargine injection	Jilin	79.0	78.1	Production
	Guangdong	90.0	95.0	Production
Dalteparin sodium injection	Jiangxi	20.0	12.0	Preclinical study
	Jiangsu	20.0	20.0	Production
Quadrivalent influenza virus split vaccine	Shanghai	50.0	450.0	Production
	Guangdong	10.0	6.0	Clinical study
Haemophilus influenzae type b conjugate vaccine	Zhejiang	17.0	13.0	Clinical study
	Jilin	200.0	140.0	Clinical study
Human fibrinogen	Shandong	35.2	36.0	Preclinical study
	Guangdong	25.0	5.0	Preclinical study
	Guangdong	40.0	35.0	Clinical study
	Shanxi	25.0	28.5	Production approval/new drug certificate
	Hunan	50.0	10.0	Clinical study
Human coagulation factor VIII	Guangdong	40.0	40.0	Production
	Guangdong	50.0	40.0	Clinical study
	Jiangxi	20.0	18.0	Clinical study
	Chongqing	33.0	28.4	Clinical study
Human prothrombin complex	Guangdong	30.0	28.0	Clinical study
	Jiangxi	20.0	20.0	Production
	Shanxi	25.0	27.7	Production approval/new drug certificate
	Sichuan	33.0	33.0	Production
	Guizhou	60.0	1.2	Preclinical study
	Chongqing	28.0	23.8	Clinical study

### 3.5 IP management new technologies/projects

More than three-quarters of the 77 enterprises included in the study have an intellectual property department (60, 77.9%) staffed with an average of four employees, and 65 (84.4%) have an established framework for IP management. Moreover, most of the companies (68, 88.3%) have proposed developmental ventures for the next 3 years with the dominant projects aimed at developing new drugs (61, 89.7%) and optimizing existing products or technology (50, 73.5%) and their quality (43, 63.2%) while also reducing the overall operational cost (36, 52.9%). The strategic plan to achieve the stated objective is mainly in-house development (28, 41.2%) but the companies also hope to collaborate with others in joint ventures (27, 39.7%). In the case of funding, more than half of the enterprises (37, 54.4%) aim to invest over 10 million yuan on their respective proposed projects, and 47 (61.0%) will also solicit external expert services as and when required (Table 5).

**TABLE 5:** New technology, intellectual property, and factors that promote the development of biopharmaceuticals.

Variable	Categories	Freq. (%)
Intellectual property department	Yes	60 (77.9)
	No	17 (22.1)
Intellectual property strategic management framework	Yes	65 (84.4)
	No	12 (15.6)
Proposals for new technologies or projects	Yes	68 (88.3)
	No	9 (11.7)
Type of proposed objectives ^a^	Develop new products	61 (89.7)
	Upgrade existing products or technologies	50 (73.5)
	Improve product quality	43 (63.2)
	Reduce manufacturing cost	36 (52.9)
	Other	2 (2.9)
Drug candidate R&D stage	Preclinical study	38 (55.9)
	Clinically approved	10 (14.7)
	Clinical study	13 (19.1)
	Production approval/New drug certificate	9 (13.2)
Planned strategy toward R&D objectives	Self-development	28 (41.2)
	Cooperative development	27 (39.7)
	Commissioned development	7 (10.3)
	Technology transfer	9 (13.2)
Proposed investment estimate in new projects or technology (Chinese yuan)	<1 million	1 (1.5)
	1–5 million	15 (22.1)
	5–10 million	15 (22.1)
	>10 million	37 (54.4)
Need for professional capacity building from outsourced agencies	Yes	47 (61.0)
	No	30 (39.0)
Expected service needing outsourcing ^a^	Policy consultation	34 (72.3)
	Legal advice	20 (42.6)
	Management consultation	11 (23.4)
	Technology assessment	28 (59.6)
	Technical brokerage	11 (23.4)
	Technology investment and financing	16 (34.0)
	Property rights transaction	10 (21.3)
	Scientific research service	23 (48.9)
	Commissioned development	27 (57.5)

a Multiple responses

### 3.6 Status of the biopharmaceutical industry in Shaanxi province

The biopharmaceutical industry in Shaanxi is challenged by a lack of the necessary local government policies that will impact on funding, innovation, and market share to motivate the producers.

Back in the 1950s, the pharmaceutical companies in Shaanxi were ranked among the top ten in China but not in recent times. This is because the provincial government did not prioritize the industry, and incentives are relatively dwarfed by those of other provinces. Subsidies in the form of soft loans and grants are difficult to access and secure. Secondly, the companies within Shaanxi province are small- and medium-scale enterprises, mainly focused on the development and marketing of Chinese traditional medicines. There is very little investment in chemical and biopharmaceutical products, and the capacity to innovate is very low [pharma company C].

Centralized public procurement serves as the main stabilizing factor and guarantees a revenue source. Nonetheless, it may result in low profit margins as a result of price control, which, coupled with difficulties in sourcing raw materials, hampers the introduction of new technology and innovative R&D in the biopharmaceutical companies.

Our biochemical product accounts for 10%-20% of our total sales; however, acquisition of raw materials has been our primary challenge. Also, even though we have most of our other products included in the national centralized procurement scheme, with large sales volumes, the price is extremely low to support R&D ventures on innovative drugs and the development of other products [pharma company A].

Our company produces and markets Citicoline Injection through the provincial centralized procurement scheme and generates a sales volume of about five million RMB annually, but we are faced with the challenge of consistency in terms of quality control due to substandard raw materials. Outsourcing for high-quality raw materials will be very costly, leading to a deficit in our investment [pharma company D].

We have two main biochemicals: ribonucleoside tablets and dextran. The sales of ribonucleoside tablets last year were less than 10 million Chinese yuan, and they are expected to reach 100 million in 4–5 years. Dextran has sales of more than 100 million. In the past, we bought raw materials from Shandong, and the adverse drug reactions were relatively large. Now that we have developed our own raw materials, the adverse drug reactions have been reduced by 90%, and patients’ responses are good. It is expected that sales will increase significantly in the future [pharma company B].

Some of the biopharmaceutical enterprises have resorted to taking measures such as streamlining their product portfolios to include the development of other products, mainly chemical drugs, reducing the production of products with a lower market share, and optimizing marketing strategies, including strengthening clinical promotion, to deal with the impact of centralized procurement.

At present, all our resources are toward R&D of new chemical drugs; we do not have plans to produce or research any biotechnology drugs [pharma company D].

We will adopt a new sales protocol that simultaneously produces and markets older medicinal products and, at the same time, focuses on biochemical pharmaceuticals. We are planning to have at most 1-2 biochemical varieties that will target markets other than the centralized procurement, thereby accumulating funds for innovative R&D [pharma company A].

We have filed an application for one of our products, an innovative drug, alprostadil liposome E1, but the process might take 2–3 years to be approved. In the meantime, we have another product that is yet to be listed in the centralized procurement system. Hence, to generate income, we are selling at the peripheral areas, focusing on clinical promotion, which is expected to have great profit margins in the future [pharma company C].

## 4 Discussion

This study explores the current status of a sizable number of biopharmaceutical companies in China in terms of their capacity to innovative and progress toward influencing the international discussion. Additionally, an evaluation of the market size of some of the biopharmaceutical products and the expenditure on R&D and drug candidates in development was done. Lastly, development in biopharmaceutical enterprises in China is not uniform across different regions and provinces; hence, we employed a detailed narrative account studying Shaanxi province to describe the progress and challenges it is presently enduring. These findings will provide a unique update to the literature and serve to inform policies on strategic interventions that need to be taken to improve the industry in provinces with a weak biopharmaceutical portfolio and in China as a whole.

### 4.1 Indicators and approach to innovation in the biopharmaceutical industry

Similar to scientific innovation in technological developments, the progress made by the biopharmaceutical sector in China is not linear but rather intricate and difficult to disaggregate. Of course, it can be argued that China stands shoulder-to-shoulder with other leading nations like the United States and European countries if the indicators taken into consideration are scientific publications and number of patents (Freire et al., 2020). Consequently, although the quantity and quality of scientific articles are predictors of scholarly progress, caution must be taken not to draw outlandish conclusions because research does not always translate to products. Likewise, the number of patents alone can be an ambiguous determinant of development and innovation (Conlé and Management, 2019). Cultivating capability is a critical construct in the progress of biopharmaceutical establishment in that it helps keep entrepreneurs afloat, attract investors, increase a company’s market share, and ensure its competitiveness in a tightly regulated industry. To make progress, a multifaceted approach has been described in the literature to include adopting high-tech manufacturing and technology transfer, skilled human resources, increased R&D spending, tailored collaboration, favorable policy frameworks, market exclusivity rights, public funding, product inclusion in public insurance schemes, and so on (Gu, 2021a; Uppal et al., 2021; Erickson et al., 2021).

### 4.2 Technology transfer: A key strategy that enhances innovation in biopharma

Many highly innovative technologies including genomic editing techniques like CRISPR were invented in universities for application in various fields. The transfer of this innovation through collaboration or for-a-fee is an ongoing practice in the science and technology community. Historically, technology transfer is seen by most nations as a public policy that helps fast track development in S&T (Graff and Sherkow, 2020). The public sector in China has supported cross-national technology transfer for decades to catch-up with the leading players in science, technology, and the biopharmaceutical sector (Hu et al., 2015; Chan et al., 2016). The most recent example that has impacted global health in developing nations is the European Union–African Union collaboration for the transfer of technology needed for the production of mRNA vaccines in six African countries (Michel, 2022). The number of companies that registered proposals of technology transfer are few, but this does not explain the whole story because our data is not representative of all biopharmaceutical enterprises in China. Even so, the practice is not as common in China as in other countries. A contributing reason for this trend that has been reported is a lack of social trust in some enterprises by the universities (Wu et al., 2021). Overcoming this impediment will require renewed engagement between academia, the public, and biopharmaceutical enterprises.

### 4.3 R&D expenditure and strategy for policy makers

The challenges faced by the biopharmaceutical industry include complexity of the products and production process, high costs, longer time-to-market, and complicated regulatory pathways, which render uncertainty in the process of developing innovative biological drugs (Rahalkar et al., 2021). Exploration of lead compounds with therapeutic potential involves their preclinical and clinical evaluation followed by a rigorous evaluation process that may last for decades incurring huge capital investments (Farid et al., 2020). Overall, more than 50% of the companies that responded to our study have a proposed investment estimated to exceed 10 million yuan in the next 3 years. R&D spending is also shown to be increasing over time, especially in companies in the eastern region (Figure 1). This finding not only signals a sustained commitment by enterprises to becoming key players in the industry but also highlights the disparity in investments by regional enterprises and governments. Narrowing the gap in R&D expenditure will need central government commitment to optimized policies that uplift provincial and regional government entities to action. This can be achieved by the provision of soft loans, grants, and tax deductions as proposed in China’s ongoing development plans (Hu et al., 2015; Zhang and Liu, 2020; Schmid and Xiong, 2021).

### 4.4 Specialized human resources: An entity of biopharmaceutical innovation

Biopharmaceutical enterprises need specialized personnel with the required qualifications to ensure sustainability in biopharmaceutical company R&D. To achieve this venture, it has been a long standing policy by the government to capacitate and support Chinese universities in cultivating local talent while providing a lucrative and competitive working environment for international experts (Hu et al., 2015). Our results showed an appreciable proportion of personnel with advanced academic degrees are employed by the enterprises included in this study. These findings are an indication that previous policies targeted at developing science and technology through education have achieved their mandate and the industry is set to grow.

### 4.5 Collaboration and its impact on biopharmaceutical development

When biopharmaceutical industries were first established, most were standalone companies. Today however, because of the resource intensiveness needed for R&D, the benefits of transnational trade and collaboration among academics, industries, government institutions, and regulators have proved to be a rewarding and cost-saving approach to achieving enterprises’ ambitions to build ecosystems that enable innovation (Tian et al., 2021). Collaboration brings together individual company strengths and experiences, enables the sharing of equipment and knowledge, and facilitates easier market entry. Smaller companies can leverage the market capitalization of larger companies and attain public trust instantly after collaboration (Moorkens et al., 2017; Erickson et al., 2021). Our results show 39% of enterprises are planning to undertake collaborative projects with other companies. The actual detail of the type of collaboration was not deduced; nonetheless, the Chinese government encouraged public–private partnerships, which have contributed in the acceleration of innovation and development of the sector (Tripathy et al., 2020). Furthermore, there are over 200 foreign biopharmaceutical companies in China and many of them are engaged in R&D locally, providing an opportunity to collaborate with their Chinese counterparts (Hu et al., 2015; Zhang and Liu, 2020). Another approach that has been adopted by the leading technology companies is the idea of open innovation. This may be complex and influence performance, as argued by Fu et al. (2019), but if tightly monitored may be rewarding to provinces in China with small and immature biopharmaceutical companies.

### 4.6 Biopharmaceutical sales in China

The sales of biopharmaceutical products are on the rise globally and also in China, as shown in our results, corroborating international trends (Moorkens et al., 2017). Previously, Chinese biopharmaceutical industries have been predicted to grow in the coming decade, yielding enormous returns (Alzahrani and Harris, 2021). According to a recent report, the global market share of biopharmaceutical products increased from 10% in 2010 to 25% in 2017 and it is expected to exceed 31% of all medicine sales by 2024, indicating a shift in need toward novel therapies (Chirmule et al., 2021; Gu, 2021a). The possible shift in demand for biologicals may be explained by an aging population in China with predicaments that are better managed by using biopharmaceuticals. Furthermore, advancement in R&D of previously untreatable diseases and the safety profile of biological drugs, an attribute of their specificity, are factors (Zhang and Liu, 2020; Schmid and Xiong, 2021). However, data from Shaanxi province show most of the sales come from government insurance and do not necessarily lead to appreciable profit margins to encourage R&D spending. To overcome these challenges, most of the companies have or are planning to invest in chemical drugs and generics targeted at export markets. The probable impact might be more reliance on profit maximization to keep the enterprises afloat than spending on innovative processes and products in the long term.

### 4.7 Leading biosimilar markets is more challenging

China and other developing countries, especially India, lead the world in terms of the manufacturing of generic medicines, enabling cost-effective medication to be accessible in most low-income nations (Uppal et al., 2021). In terms of biopharmaceuticals, biosimilars are the generic counterpart. A thoughtful question one may pose is, will China be a leading producer of biosimilars as it is in the generic drug market? Obviously, it is difficult to say because, unlike generics drugs that are chemical entities, biopharmaceutical production takes more than just having the production protocols or leveraging patent expiration of originator brands. The manufacturing of biosimilars requires complex production processes, larger investment ventures, and high-quality and specific raw materials, and the approval process for biosimilars is extensive and rigorous to ensure patient safety (Chirmule et al., 2021; Uppal et al., 2021). The current findings show a growing challenge in the development of biopharmaceutical enterprises in Shaanxi province and the constraints they endure in the sourcing of quality raw materials. The resulting effect is a polarized risk to any investment and patients, which might explain the status of the sector in the province.

### 4.8 Study strength and limitations

Our study has limitations that should be considered when interpreting the findings. Firstly, a non-probabilistic sampling procedure was used to recruit participants, which renders the findings vulnerable to bias; notwithstanding that, considering the state of the pandemic and vastness of the pharmaceutical establishment in China, we believe our approach is the most feasible. Secondly, even though a mixed-method approach was used, the data were collected at one time point; data that support trend analysis of the indicators evaluated will be more impactful. The results cannot be generalized for the whole of China nor for the whole of Shaanxi province because the survey data are non-representative of biopharmaceutical companies nationwide; moreover, the amount of qualitative data is small and includes only a few responses from Shaanxi province. The opinion of representative pharmaceutical companies from other regions will be insightful. Moreover, there is a need for broader and extensive research on the subject nationwide to incorporate more regions with weaker biopharmaceutical innovation and development that will warrant a general targeted policy formulation. There is no clear explicit model of determining innovation in the sector because of it is ambiguous and dependent on many factors; hence, this study focuses on what may be regarded as some indicators, including qualified personnel, infrastructure, funding, market capitalization, and local policies in the case of Shaanxi, which are not exhaustive. Follow-up studies should engage other parameters that have been reported in the literature as blueprints in evaluating biopharmaceutical innovation and development.

## 5 Conclusion

The biopharmaceutical industry in China is relatively large and on a trajectory toward continued development and achieving its aim of being a global leader in the sector. As per our results, it can be deduced that there is an increase in demand for biopharmaceutical products as indicated by the increase in sales. Furthermore, most companies have skilled and educated members in their enterprise and funding for R&D keeps increasing, which are key indicators that impact innovation and development. However, innovation and development capacity of the sector needs to be further improved, especially in the western region, as shown from the findings from Shaanxi province, where research capabilities and production capacity are relatively weak. Therefore, the government and policy makers must continue to support policies that promote innovation of the industry nationally; this may include continued scaling up of public funding for the training of biomedical scientists, an increase in R&D of new drugs, and prioritizing greater policy preferences in regions with weaker industries to sustain the gains made in past decades. At the provincial level, the provision of low interest loans, tax deductions for investments in innovative and orphan drugs, and marketing and patent policies that do not discourage R&D in the sector might lead to improvements in the industry.

## Data Availability

The raw data supporting the conclusion of this article will be made available by the authors, without undue reservation.
