# Supplementary material for: Current situation and future development of the biopharmaceutical industry in China: A mixed-method study
Source: Front Pharmacol. 2022 Aug 22;13:911165. doi: 10.3389/fphar.2022.911165 (PMC9442039; doi:10.3389/fphar.2022.911165)
Supplement: Supplementary file 1 [file DataSheet1.docx]

**Appendix** 1: Questionnaire

| Company’s name: | | | | | | | | | | | | |
| --- | --- | --- | --- | --- | --- | --- | --- | --- | --- | --- | --- | --- |
| Whether it is a high-tech enterprise:　□Yes □No | | | | | | | | | | | | |
| The total number of existing employees: | | | | | | | The total number of R&D personnel: | | | | | |
| Whether to set up an independent R&D department: □Yes □No | | | | | | | | | | | | |
| Composition of R&D personnel | | | | | | | | | | | | |
| Doctor：……..  Master：……. | | | | | Senior title:  Intermediate title: | | | | | | | |
| **Product (Chinese yuan)** | | | | | | | | | | | | |
| Names of the top three biotech drugs/biochemical drugs in sales | | | | 2018 | | | | 2019 | | | | 2020 |
|  | | | |  | | | |  | | | |  |
|  | | | |  | | | |  | | | |  |
|  | | | |  | | | |  | | | |  |
| Total | | | |  | | | |  | | | |  |
| **Annual R&D expenditures (Chinese yuan)** | | | | | | | | | | | | |
| Year | Expenditure | Sources | | | | | | | | | | |
|  |  | Private capital | | Government funding | | Bank loan | | | Venture capital | | Other | |
| 2018 |  |  | |  | |  | | |  | |  | |
| 2019 |  |  | |  | |  | | |  | |  | |
| 2020 |  |  | |  | |  | | |  | |  | |
| **New technology projects in development in the past 3 years** | | | | | | | | | | | | |
| Category | | | Project’s name | | | | | | | Investment (Chinese yuan) | | |
| Cooperative development project | | |  | | | | | | |  | | |
| Commissioned development project | | |  | | | | | | |  | | |
| Technology transfer project | | |  | | | | | | |  | | |
| Enterprise independent project | | |  | | | | | | |  | | |

| **New products in development in the past 5 years** | | | |
| --- | --- | --- | --- |
| Product’s name | Development stage | Planned investment (Chinese yuan) | Total expenditure (Chinese yuan) |
|  | □ Preclinical studies  □ clinically approved  □ clinical studies  □ production approval/new drug certificate  □ production |  |  |
|  | □ Preclinical studies  □ clinically approved  □ clinical studies  □ production approval/new drug certificate  □ production |  |  |
| **Patent** | | | |
| Does the company have intellectual property departments or personnel: □Yes (number: ) □No | | | |
| Does the company have an intellectual property strategy or plan: □Yes □No | | | |
| Will, there be new technology (project) venture in the next 3 years: □Yes □No  **(If you choose "Yes", please fill in the following)**  Purpose (multiple choices available)：□Product or technology upgrade □decline production cost □develop new product □improve product quality □Others  The research stage of the demanding project:  □Preclinical studies □clinically approved □clinical studies □production approval/new drug certificate  Solution:□Self-developed □Co-development □Commissioned development □Technology transfer  Planned investment：□<1 million □1-5 million □5-10 million □>10million | | | |
| Are you willing to solicit capacity-building services provided by intermediary agencies?  □Yes □No  **(If you choose "Yes", please fill in the following)**  Expected services (multiple choices available): □Policy consultation □Legal advice □Management consulting □Technology assessment □Technical broker □Technology investment and financing　□Property rights transaction □Scientific research service □Commissioned development | | | |

**Appendix 2:** Interview guide

1. What is the biggest impediment to the development of biopharmaceutical companies in Shaanxi Province?
2. What are the opportunities and challenges does the current national drug negotiations bring to your biopharmaceutical companies?
3. With the implementation of the national centralized procurement policy, what changes have taken place in the sales model of biopharmaceutical companies?
4. The implementation of policies such as centralized drug procurement and national drug negotiation has had a huge impact on the development of biopharmaceutical companies. How do you view the survival and development difficulties faced by biopharmaceutical companies in the short term and long-term sustainable development?
5. The function of medical insurance is to "guarantee basics." Therefore, not all innovative drugs have the opportunity or can enter medical insurance. What kind of innovative drugs do you think should be included in the medical insurance drug list first?
6. "The list of medical insurance medications supports the clinical value of drugs, rather than simply supporting innovation." What do you think of this sentence?
